# Supplementary material for: Identification of photoperiod-induced specific miRNAs in the adrenal glands of Sunite sheep (Ovis aries)
Source: Front Vet Sci. 2022 Jul 22;9:888207. doi: 10.3389/fvets.2022.888207 (PMC9354845; doi:10.3389/fvets.2022.888207)
Supplement: Supplementary file 1 [file Data_Sheet_1.ZIP › Supplementary file/miRDeep2 file.zip!/miRDeep2 file/Novel_Structure/LP42A1/Novel_63.pdf]

Provisional ID	: NC_019459.2_5834
Score total	: 1.2
Score for star read(s)	: -1.3
Score for read counts	: 0
Score for mfe	: 0.9
Score for randfold	: 1.6
Score for cons. seed	:
Total read count	: 18
Mature read count	: 18
Loop read count	: 0
Star read count	: 0

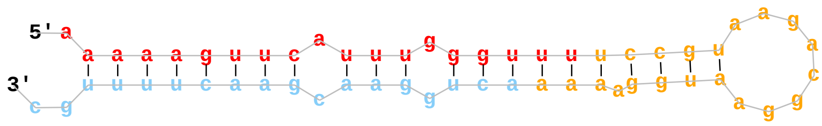

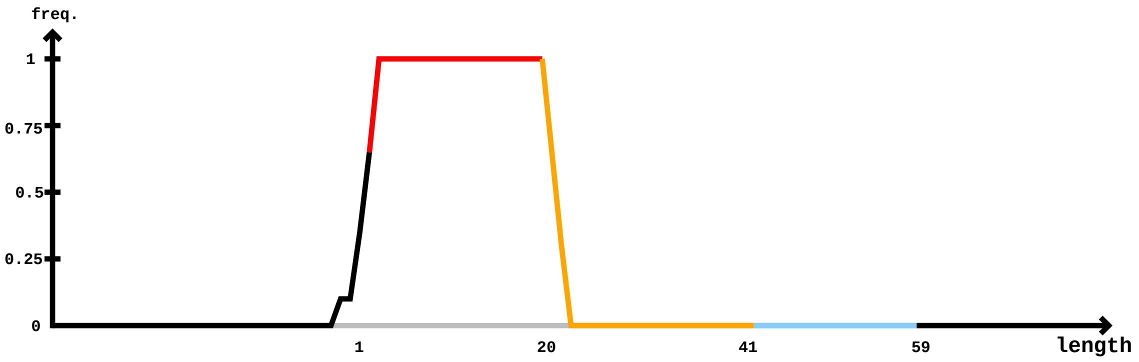

Mature Star

```
5'- ccucuggcucuaucauugucgugaguuggucaaaaaguucauuuggguuuuccguaagacggaauggaaaaacuggaacgaacuuuugcccaauccaguacacagccu   -3'    exp
    .....((((.......(((..((.(((((.(((((.((((.(((.((((((((((........)))).)))))).))).))))))))).))))).))..))).)))).    reads     mm     sample
    ............................gucaaaaaAuucauuuggguuu..........................................................    2         1      seq
    ..............................caaaaaguucGuuuggguuuu.........................................................    5         1      seq
    ...............................aaaaaguucGuuuggguuu..........................................................    6         1      seq
    ................................aaaaguucauuAggguuuu.........................................................    1         1      seq
    ................................aaaaguucGuuuggguuuuc........................................................    6         1      seq
```
